# Supplementary material for: Effects of steroid therapy in patients with severe fever with Thrombocytopenia syndrome: A multicenter clinical cohort study
Source: PLoS Negl Trop Dis. 2021 Feb 19;15(2):e0009128. doi: 10.1371/journal.pntd.0009128 (PMC7928499; doi:10.1371/journal.pntd.0009128)
Supplement: S1 Table — (DOCX) [file pntd.0009128.s001.docx]

**S1 Table. Additional Information of General and Clinical Characteristics of Patients with SFTS in the Non-Fatal and Fatal Groups (2013–2017)**

|  | Non-Fatal | | Fatal^a^ | | Total | |  |
| --- | --- | --- | --- | --- | --- | --- | --- |
| Characteristics | n = 109 | | n = 33 | | N = 142 | | *P* Value^b^ |
| Occupation |  |  |  |  |  |  |  |
| Agriculture | 44 | (40.4) | 11 | (33.3) | 55 | (38.7) | .544 |
| Tick bite |  |  |  |  |  |  |  |
| Memory of tick bite^c^ | 26 | (26.3) | 5 | (16.1) | 31 | (23.8) | .248 |
| Presence of bite wound^d^ | 35 | (34.3) | 12 | (41.4) | 47 | (35.9) | .484 |
| Comorbidity, total | 64 | (58.7) | 24 | (72.7) | 88 | (62.0) | .146 |
| DM | 23 | (21.1) | 10 | (30.3) | 33 | (23.2) | .273 |
| HTN | 39 | (35.8) | 15 | (45.5) | 54 | (38.0) | .316 |
| CVA | 5 | (4.6) | 4 | (12.1) | 9 | (6.3) | .213 |
| CHF | 2 | (1.8) | 0 | (0.0) | 2 | (1.4) | N/A |
| CLD | 3 | (2.8) | 0 | (0.0) | 3 | (2.1) | N/A |
| CKD | 1 | (0.9) | 1 | (3.0) | 2 | (1.4) | .412 |
| Asthma | 0 | (0.0) | 1 | (3.0) | 1 | (0.7) | N/A |
| COPD | 2 | (1.8) | 1 | (3.0) | 3 | (2.1) | .551 |
| Solid tumor | 3 | (2.8) | 1 | (3.0) | 4 | (2.8) | 1.000 |
| Data are presented as no. (%) unless otherwise indicated.  Abbreviations: CHF, congestive heart failure; CKD, chronic kidney diseases; CLD, chronic liver disease; COPD, chronic obstructive pulmonary disease; CVA, cerebrovascular accident; DM, diabetes mellitus; HTN, hypertension; N/A, not available.  ^a^ Of 29 patients died within 30-day from admission, and 1 patients died over 30-day from admission. Missing data of 30-day survival time was 3 because these were out-hospital death.  ^b^ Analysis using a Chi-square test, or a Fisher's exact test.  ^c^ Missing data: n = 11.  ^d^ Missing data: n = 12. | | | | | | | |
