# Supplementary material for: Effects of steroid therapy in patients with severe fever with Thrombocytopenia syndrome: A multicenter clinical cohort study
Source: PLoS Negl Trop Dis. 2021 Feb 19;15(2):e0009128. doi: 10.1371/journal.pntd.0009128 (PMC7928499; doi:10.1371/journal.pntd.0009128)
Supplement: S2 Table — (DOCX) [file pntd.0009128.s002.docx]

**S2 Table. Additional Information on the Clinical Characteristics of Patients with SFTS in the Steroid and Non-Steroid Groups (2013–2017)**

|  | Non-Steroid | | Steroid | | Total | |  |
| --- | --- | --- | --- | --- | --- | --- | --- |
| Characteristics | n = 84 | | n = 58 | | N = 142 | | *P* Value^a^ |
| Comorbidity, total | 50 | (59.5) | 38 | (65.5) | 88 | (62.0) | .470 |
| DM | 15 | (17.9) | 18 | (31.0) | 33 | (23.2) | .068 |
| HTN | 30 | (35.7) | 24 | (41.4) | 54 | (38.0) | .494 |
| CVA | 3 | (3.6) | 6 | (10.3) | 9 | (6.3) | .103 |
| CHF | 2 | (3.4) | 0 | (0.0) | 2 | (1.4) | N/A |
| CLD | 1 | (1.2) | 2 | (3.4) | 3 | (2.1) | .567 |
| CKD | 1 | (1.2) | 1 | (1.7) | 2 | (1.4) | 1.000 |
| Asthma | 0 | (0.0) | 1 | (1.7) | 1 | (0.7) | N/A |
| COPD | 3 | (3.6) | 0 | (0.0) | 3 | (2.1) | N/A |
| Solid tumor | 3 | (3.6) | 1 | (1.7) | 4 | (2.8) | .645 |
| Initial clinical manifestation^b^ |  |  |  |  |  |  |  |
| Systemic |  |  |  |  |  |  |  |
| Fever | 76 | (91.6) | 45 | (78.9) | 121 | (86.4) | .018 |
| Chills | 54 | (65.1) | 27 | (47.4) | 81 | (57.9) | .033 |
| Myalgia | 42 | (50.6) | 18 | (31.6) | 60 | (42.9) | .038 |
| Arthralgia | 4 | (4.8) | 2 | (3.5) | 6 | (4.3) | 1.000 |
| Fatigue | 26 | (31.3) | 8 | (14.0) | 34 | (24.3) | .023 |
| Back pain | 8 | (9.6) | 1 | (1.8) | 9 | (6.4) | .080 |
| Gastrointestinal | 55 | (66.3) | 39 | (68.4) | 94 | (67.1) | .709 |
| Anorexia | 22 | (26.5) | 21 | (36.8) | 43 | (30.7) | .143 |
| Nausea | 27 | (32.5) | 11 | (19.3) | 38 | (27.1) | .083 |
| Vomiting | 15 | (18.1) | 10 | (17.5) | 25 | (17.9) | .918 |
| Diarrhea | 20 | (24.1) | 22 | (38.6) | 42 | (30.0) | .067 |
| Abdominal pain | 20 | (24.1) | 11 | (19.3) | 31 | (22.1) | .518 |
| Abdominal tenderness | 6 | (7.2) | 4 | (7.0) | 10 | (7.1) | 1.000 |
| Central nervous system | 40 | (48.2) | 29 | (50.9) | 69 | (49.3) | .887 |
| Headache | 25 | (30.1) | 14 | (24.6) | 39 | (27.9) | .506 |
| Dizziness | 9 | (10.8) | 8 | (14.0) | 17 | (12.1) | .632 |
| Neck stiffness | 2 | (2.4) | 3 | (5.3) | 5 | (3.6) | .648 |
| Altered mentation | 15 | (18.1) | 14 | (24.6) | 29 | (20.7) | .424 |
| Data are presented as no. (%) unless otherwise indicated.  Abbreviations: CHF, congestive heart failure; CKD, chronic kidney diseases; CLD, chronic liver disease; COPD, chronic obstructive pulmonary disease; CVA, cerebrovascular accident; DM, diabetes mellitus; HTN, hypertension; N/A, not available.  ^a^ Analysis using a Chi-square test, or a Fisher's exact test.  ^b^ Missing data = 2 (non-steroid, n = 1; steroid, n = 1). | | | | | | | |
