# Supplementary material for: Effects of steroid therapy in patients with severe fever with Thrombocytopenia syndrome: A multicenter clinical cohort study
Source: PLoS Negl Trop Dis. 2021 Feb 19;15(2):e0009128. doi: 10.1371/journal.pntd.0009128 (PMC7928499; doi:10.1371/journal.pntd.0009128)
Supplement: Attachment — Submitted filename: Response letter PNTD-D-20-01574.docx [file pntd.0009128.s003.docx]

16^th^ October 2020

A. Desiree LaBeaud - Deputy Editor
Anita K. McElroy - Associate Editor
*PLOS Neglected Tropical Diseases*

Dear Editors,

I, along with my co-authors, would like to re-submit the attached manuscript entitled “Effects of Steroid Therapy in Patients with Severe Fever with Thrombocytopenia Syndrome: A Multicenter Clinical Cohort Study”. The manuscript ID is **PNTD-D-20-01574**.

We would like to thank all the reviewers and editors for taking the time to review and are grateful for their constructive comments and valuable improvements to our manuscript. In response to the reviewer’s suggestions and questions, we have addressed all the raised concerns. We believe that by making these changes the quality of the manuscript has been substantially improved. Please see below, in bold, for a point-by-point response to reviewers’ comments and concerns. All page numbers refer to the revised manuscript file with track changes.

With best wishes

**Dong-Min Kim**

Department of Internal Medicine, College of Medicine, Chosun University

588 Seosuk-dong, Dong-gu, Gwangju 61453, Republic of Korea

Tel: 82-62-220-3108; Fax: 82-62-234-9653

**Methods**

Reviewer #1: I have no major concerns about the methods. I do think that the sample size is somewhat small to use for propensity score matching, but I understand that patient volume does not currently allow greater numbers, and the authors are trying to achieve a comparison of the treatment between similar groups.
**Author Response: We thank reviewer for the remarks on methods. We have tried to provide concrete evidence as possible.**

Reviewer #2: See Summary and General Comments

Other comments:
Pg 5, line 109: what were the specific inclusion and exclusion criteria? Was hospitalization an inclusion criterion?
**Author Response:** **The inclusion criteria were that 1) patients who admitted to hospitals from 2013 to 2017, and 2) those who confirmed SFTS by molecular or serology test for SFTSV conducted by the KCDC, Korea Institute of Health and Environment, or Chosun University Hospital. The exclusion criteria was of patients with co-infection for any other pathogen besides SFTSV (page 5, Line 110).**

Pg 5, line 116: What other pathogens were routinely tested for?
**Author Response: There were different pathogen types tested by routines between hospitals. However, tests were conducted to identify the pathogens of tick-borne diseases or vector-borne diseases such as scrub typhus(Orientia tsutsugamushi), leptospirosis(Leptospira), and hemorrhagic fever with renal syndrome(Hantaan virus) in common with each hospitals. Of the 142 participants in this study, 51 patients whose samples were available were tested for scrub typhus and HFRS by PCR, and all of them were the results of negative.**

Pg 6, line 131: It would be useful to list the components of the APACHE II score (possibly in a supplementary table)
**Author Response: Thank you for this suggestion. A supplementary table has been added (S1 Table).**

Reviewer #3: The objective of this study was to evaluate the effect of steroids in the treatment of patients hospitalized with SFTSV. The study presented was a multicenter retrospective cohort study, which is an appropriate study design to address this clinical question, given the relative infrequency of infection with this virus. The population under study is well-described, and is sufficiently large for the authors to identify statistically significant effects associated with steroid treatment.
Of critical concern in the interpretation of retrospective cohort studies is the potential for confounding. In this study, a possible alternative explanation for the author’s findings is that the population receiving steroid treatment is different than the untreated population in some way, for instance, in severity of disease, timing after symptom onset, prominence of respiratory symptoms, etc. Propensity matching is an appropriate method to control for some of these differences, however, concerns regarding residual confounding remain.
**Author Response: We appreciate the positive feedback related to study design, data set and methods. As we explained the PS matching variable selection process in the method, in order to minimize residual confounding remain, various variables including severity of disease, timing after symptom onset, and prominence of respiratory symptoms were analyzed to confirm confounding factors and then we have selected the final matching variable. As this study is a retrospective study and not a randomized control trial, there is some potential for confounding though. We added the study limitation in discussion section.**

Additional comments regarding methods:
Line 112 – 115: The authors state that the diagnosis of SFTS is made by a combination of molecular methods (PCR/RTPCR) and serology. The sensitivity and specificity of these tests are presumably quite different; therefore, it would be useful to specify how many study participants were diagnosed via molecular methods v. serology.
**Author Response: 142 participants were diagnosed via molecular methods, and 12 participants were also diagnosed via more than 4 fold IgG antibody increase using IFA in duplicate.**

Line 130: To assess differences in 30d mortality, the authors state that they used a Cox regression model adjusted for initial APACHE score and hospitalization within seven days of symptom onset. Why were these variables chosen? Table 2 suggests that there is a small (but significant) difference in APACHE scores between those participants who received steroids and those who did not, but the variable entitled ‘Symptom onset to admission’ in Table 2 was the same between the two groups. Does the description in the methods ‘hospitalization within 7 days of symptom onset’ represent a categorical variable derived from the continuous variable ‘Symptom onset to admission’? This should be clearer.
**Authors Response: We determined that it was necessary to analyze confounders such as severity of disease and timing after symptom onset among several variables affecting treatment effect at 30-day mortality although there were no statistical differences. In our dataset, we selected the ‘initial APACHE II score’ variable representing the severity of disease, and the ‘hospitalization within 7 days after symptom onset’ variable representing timing after symptom onset identified as common confounders of patients applying ribavirin, steroids, IVIG, and plasmapheresis. The ‘hospitalization within 7 days after symptom onset’ variable was categorized from continuous variable as ‘Days of symptom onset to admission’ because this period was the critical stage in SFTS progression in the previous study[12].**

**(12. Gai ZT, Zhang Y, Liang MF, et al. Clinical progress and risk factors for death in severe fever with thrombocytopenia syndrome patients. J Infect Dis 2012; 206:1095-102.)**

Line 140: What do ‘Initial altered mental state’ and ‘initial intensive care unit (ICU) admission’ mean?

**Author Response: ‘Initial altered mental state’ means altered mentation between onset and admission. ‘Initial intensive care unit admission’ means an intensive care unit admission at the beginning of hospitalization. To clarify it we added the definition as a footnote in Table 6.**

Line 213: Were all of the variables used for univariate analysis included in the multivariate analysis in Table 4?
**Author Response: Yes, all of the variables used for univariate analysis in Table 4 were included in the multivariate analysis. To reduce confusion, we have added the information of the variables included in the multivariate model as a footnote in Table 4.**

Line 221: What variables were included in the multivariate analysis presented in Table 5? Are these variables the same as those included in the multivariate analysis in Table 4? This should be clearer.
**Author Response: The variables included in the multivariate analysis were treatment variables such as prior antibiotic treatment, ribavirin, steroids, IVIG, and plasmapheresis, and covariate variables such as age, sex, initial APACHE II score and symptom onset to admission within 7 days. We have revised this information and added it as a footnote in Table 5.**

--------------------

**Results**

Reviewer #1: Tables 1 and 2 are especially long and more appropriate as supplemental tables. Both include information that I am not sure is necessary for the purposes of the paper, such as tick bit and occupation info in table 1 and two classifications each for elevated AST and ALT. Clinical manifestations could perhaps be reduced to the system and the most important symptoms, such as altered mentation and GCS for the central nervous system.
**Author Response: Thank you for good comments. We tried to described related variables of table 1 and 2 which were used to check the risk factors of 30-day mortality in table 4. As suggested by the reviewer, we have represented variables in Table1, 2, S2 Table and S3 Table.**

It is also unclear to me whether ICU admission in these tables refers to any point in the hospital course or if that is describing where they were initially admitted. My presumption is the former, but then this means that 15% of patients who died were never admitted to the ICU.
**Author Response: Thank you for your comments. ‘ICU admission’ in Tables 1 and 2 means ICU admission during hospitalization. We have tried to make these confusing terms more clear, so please refer to tables 1 and 2.**

The information regarding steroid administration is extremely vague, simply giving a range of doses received without any additional information other than median duration. A dose of dexamethasone 5 mg or methylpred 40 mg is quite different from dexamethasone 30 mg or methylpred 1000 mg. Knowing the distribution in doses would help the reader frame this analysis appropriately. The ideal analysis would stratify by steroid dosing in some way, but it is unlikely there are sufficient numbers for this. **Author Response: Thank you for pointing out important and valuable points, and we agree with the reviewer’s advice. This study was retrospective study, physicians prescribed steroid of their own judgement, we just collected the information on steroid treatments. It was difficult to include enough samples for stratified subgroup analysis in the retrospective study. In future research, it will be important to extend the period and generate more concrete evidence through subgroup analysis or prospective study with more organizations.**

Regarding Table 3, the authors should clarify how values were selected if there was more than 1 in that timeframe? For instance, there were no doubt multiple body temperature measurements in the 48 hours before and after treatment. Did they select the highest temperature, an average of the values, or base in on time criteria? Likely some patients had labs performed more than once in that period as well.
**Author Response: We collected clinical and laboratory data on specified dates, and collected the most severe value if measured more than once on that date.**

Lastly, additional information on when patients were admitted to the ICU and developed complications in relation to timing of steroids would be appreciated. If most patients received steroids prior to ICU admission, that frames this differently than if most patients received steroids after worsening and transfer to the ICU. The authors note that mechanical ventilation was required more often and DIC was more common in the steroid group. However, it is not at all clear whether these complications developed before or after steroid administration. Without this information, it is very easy to imagine (particularly when considering the late steroid group) that steroids were actually given because of these complications.
**Author Response: We appreciate for pointing out important things. In our dataset, 19 patients received steroids without ICU admission, and 39 patients were treated with steroids among ICU patients. Of the 39 patients, 10 were given steroids before admission to the ICU, and 29 were given steroids after admission to the ICU.**

**Among the steroid-administered patients with ICU hospitalization, 21 patients had undergone ventilator treatment, of which 9/21 patients (42.9%) started ventilator treatment from before steroid treatment to start of treatment, and 12/21patients(57.1%) who started ventilator treatment after starting steroid treatment. As this study had difficulty in determining the timing of complications after steroid treatment due to the limitation of retrospective study, it supported that a prospective study was necessary in the future.**

Reviewer #2: It would be useful to add the total number of patients tested for SFTS during the time period.
Were all eligible patients included? If not, authors need to explain why not.
**Author Response: Thank you for your valuable comments. We have updated the figure 1 to add the total number of patients tested for SFTS. All eligible patients were included in this study.**

Table 1: the numbers of evaluable cases of “memory of tick bite” (n=130) and “presence of bite wound” (n=131) should be added to indicate that some were missing. The statistical test for comparing respiration rates should be checked—the difference does not appear significant although p=0.013
**Author Response: Thank you for your comments. We have added information about the number of missing data of it and reorganized it in S2 Table as following the other reviewer’s suggestion. We have checked again at the data of respiration rates, and the results of analysis was correct. The distributions of respiration rates were different between non-fatal and fatal group as you can see IQR of them. We used analysis non-parametric method to analyze the difference of respiration rates between groups.**

Pg 10, line 177: Steroid therapy: did any local hospital-based guidelines exist that may explain why some patients received steroids whereas others didn’t? Did rates of steroid use differ between hospitals? What type of steroid was used for patients who did not receive dexamethasone or methylprednisolone (n=9)? The authors state that 58 patients received steroids but the numbers who received steroids alone (n=19), steroids + ribavirin (n=20), steroids + IVIG (n=25) and steroids + plasmapheresis (n=15) exceeds 58.
**Author Response: Effective treatment for SFTS has not been identified so far, there were no specific guidelines for treatment such as steroid therapy. Therefore, steroid therapy was used differently on physicians, and the number of steroid group was small, making it difficult to perform stratification analysis by subgroups. Other types of steroids not mentioned in the manuscript were prednisolone (n=4) and hydrocortisone (n=10). The reason the total number was not 58 is that there were patients who had given more 2 types of steroids. The number of patients receiving one type of steroid was 53 (dexamethasone = 31, prednisolone = 2, methylprednisolone = 12, hydrocortisone = 8), and the number of patients receiving two types of steroid was 5 (dexamethasone + hydrocortisone = 1, dexamethasone + methylprednisolone =1, prednisolone+methylprednisolone = 2, methylprednisolone + hydrocortisone = 1). Also, the total number of steroids and combined treatments exceeds 58 because some patients received three combined treatments in duplicate.**

Table 2:
GCS in each group appears to be very similar but p=0.008; stats should be checked
**Author Response: Thank you for your comments. We have checked again at the data of GCS score, and the results of analysis was correct. As you can see IQR of them, the distributions of GCS score were different between non-steroid and steroid groups. We used analysis non-parametric method to analyze the difference of GCS score between groups.**

Table 3:
It is not clear why the numbers of patients included in the “before vs after” steroid treatment groups are different because the authors stated on pg 12, line 198 that patients who “underwent laboratory testing BOTH pre- and post-steroid treatment are shown”. Therefore, it seems the numbers should be the same in each group.
**Author Response: Thank you for pointing out by the reviewer. In our dataset, which was a retrospective study and used to compare 48 hours before and after steroid treatment, 41 out of 58 patients who received steroid treatment had the results of laboratory test, but not all items of the test were performed, so the number of each item were different.**

Pg 19, line 268: there is a typo—it should read “in the NON-STEROID group than in the STEROID group” not vice versa
**Author Response: Thank you for your comments, we have revised as per your recommendation.**

Table 7:
It is unnecessary to include n for each variable since they are all the same—n can be included as a footnote.
**Author Response: Thank you for your comments, we have revised as per your recommendation. In addition, in order to reinforce evidence and eliminate residual confounder(according to the comments of other reviewer), we have analyzed complications between non-steroid and steroid groups using the dataset after propensity score matching and corrected this data in table 7.**

The figures are of sufficient quality for clarity

**Author Response: Thank you for your remarks on figures.**

Reviewer #3: The presented analysis matches the analysis plan. Overall, the results were clearly presented, and support the author’s conclusions. However, there are several inconsistencies and omissions than should be addressed before the paper is published. In particular, it is disconcerting that the analyses presented in Figures 2 and 3 include different numbers of participants than from each other, or from the table outlining the propensity-matched data set in Table 6. Given the relatively small sample size, the most rigorous approach would be to conduct the analyses presented here using the same propensity matched dataset.
**Author Response: Thanks for the valuable comment from the reviewer. There were different numbers of participants between Figure 2 and 3 because there were missing data of APACHE II score as a result of analysis using the same propensity score matching dataset. In this regard, as the reviewer’s comments, we explained the reasons for missing data as footnotes in figures 2 and 3.**

Additional comments regarding results:
Line 149: The results state that of 142 patients, 33 died. Were these deaths captured within a certain period of time after hospitalization? Are these in-hospital deaths? Deaths within 30d of admission? Fatal should be better defined here and in Table 1.
**Author Response: Of the 33 deaths, in-hospital deaths were 29 and out-hospital deaths were hopeless discharge, with 4. One of the deceased died more than 30 days after hospitalization, and 3 out of 4 hopeless discharge had no date of death, so the 30-d survival time was censored. All else died within 30 days. According to the reviewer's comment, we also added the data on the mortality rate within 30 days as a footnote to Table 1.**

Line 173: These symptoms are noted as ‘initial major symptoms’. Does that mean that only those patients who had fever, chills, etc., at the time of presentation are counted as positive? If these symptoms developed later, are patients still counted as positive?
**Author Response: These symptoms that appeared from the onset to the time of admission of hospitals were counted by checking the presentation. Symptoms after hospitalization were not included. The reason we used only these data was that we expected various confounders influencing the treatment effect, so we used these variables indicating symptoms confirmed the presence or absence at the time of initial hospitalization, before treatment started.**

Line 176: Why are fatal cases less likely to have fever, chills, and myalgias than nonfatal cases? The higher incidence of altered mental status may mean that patients ware less likely to report subjective symptoms (chills, myalgias), but the lower rate of fever raises concerns that patients who end up dying are already different at the time of presentation. Are fatal cases further along in their disease course than nonfatal cases? Are they already receiving some intervention that masks fevers, such as steroids or CRRT? This finding raises concerns about underlying confounding between fatal and nonfatal cases that might bias the analysis of steroids and other interventions – if fatal cases were already sicker at the time of presentation, and are started on CRRT or steroids more rapidly, then the apparent reduction in 30d mortality may reflect confounding rather than effects attributable to an intervention.
**Author Response: We guess that the higher the incidence of mental change in fatal cases, the less likely they were to express initial subjective symptoms. Therefore, in order to consider the underlying confounding stated by the reviewer, we tried to confirm the confounder by analyzing objective data on initial V/S and initial lab result as data in addition to the patient's initial subjective symptoms. In the proportions of initial treatments of CRRT or steroids, there was no difference statistically between groups with and without fever or fatal and non-fatal groups between onset to admission, so the treatments do not seem to mask fevers (Initial steroid (non-fatal/fatal: n=3/109, 3/33, p=.138), Initial CRRT (non-fata/fatal: n=3/109, 3/33, p=.138) .**

**In addition, we founded that there was a difference between fatal and non-fatal groups in the initial APACHE II score variable, and it was a confounder which was considered of further progression of the disease process between fatal and non-fatal groups.**

**Therefore, it is considered that the reflection of confounding was sufficiently silenced because variables that check severity such as initial APACHE II score were also included as PS matching variables.**

Table 177: Of the 58 patients who received steroid therapy, 24 died. This information is not currently available in Table 1 or Table 2. It should be in one place or the other, preferably both. Would include a line in Table 1 denoting steroid treatment, and a line in Table 2 denoting mortality.
**Author Response: Thank you for your comments, we have revised as per your recommendation.**

Line 177 – 180: Information about steroid dosing should be included in Table 2.
**Author Response: Thank you for your comments, we have included the data of steroid dosing in Table 2 as per your recommendation.**

Line 180 – 183: This information is included almost verbatim in the next paragraph. It doesn’t need to be repeated.

**Author Response: Thank you for your comments, we have eliminated the sentence as per your recommendation.**

Line 212: How were these variables selected for univariate analysis? Were other variables considered?

**Author Response:** **In 142 patients, we conducted univariate analysis with variables in table 4 and other variables, and we selected variables as p value < .1 in the results of univariate analysis to conduct multivariate analysis following table 4.**

Line 233 – 238: Does this information about 30d survival appear elsewhere in the study?
**Author Response: We analyzed more specific data about Figure 2A, but we didn’t show tables or figures about this information.**

Table 6: Why did the number of participants go from 56 to 58 after propensity matching? Might be helpful to know why those two participants were excluded.

**Author Response: The number of patients in the steroid group in the first dataset was 58. After propensity score matching, in the process of selecting a control group that matches the steroid group tendency, two patients in the steroid group were also eliminated because there was no control group that matched the steroid group tendency.**

Figure 2B: Why were 7 patients excluded (n=135 instead of n = 142)? Should include a footnote explaining the exclusions.

**Author Response: 7 patients were missed 30 day survival time because we couldn’t exactly figure 30 day survival time such as hopeless discharge and discharge of no reason not for clinical reasons. As the reviewer’s suggestion, we have added this information as a footnote on Figure 2.**

Figure 2C: Why does the non-steroid group now have 52 participants instead of 56 after propensity matching? Is this figure legend incorrect, or is Table 6 incorrect?

**Author Response: Since subjects with the missing data of 30 day survival time from the control group were exclude after propensity score matching, 52 out of 56 non-steroid groups were analyzed. We have added this information on Figure 2.**

Figure 3A: Why does this analysis have 120 instead of 135 participants? Again, if patients are being excluded, should create a footnote detailing why.
**Author Response: In the case of Figure 3A, out of a total of 142 patients as data before propensity score matching, 22 patients with missing data for 30-day survival time and initial APACHE II score were excluded. We have added this information as a footnote on figure 3 as per your recommendation.**

Line 286: Would be careful with chi-square P values for groups that have fewer than 5 members; it is difficult to conclude that the frequency of DIC was actually significant given small sample size.

**Author Response: Thank you for the good point. We have deleted the related sentence.**

Table 7: Would omit columns for ‘N’, since these are the same for all values; doesn’t add anything to table.

**Author Response: Thank you for your comments, we have revised as per your recommendation. In addition, in order to reinforce evidence and eliminate residual confounder(according to the comments of other reviewer), we have analyzed complications between non-steroid and steroid groups using the dataset after propensity score matching and corrected this data in table 7.**


--------------------

**Conclusions**


Reviewer #1: Pending response to the issues above, the conclusion by the authors may be too strong. In the last paragraph, they state that they observed increased complications after steroid therapy. They may very well have observed this, but this is not clearly demonstrated by the data presented in the paper. They otherwise are reasonably cautious about whether steroids may cause increased mortality in SFTS. Depending on the distribution of steroid doses, I believe the lack of similar steroid dosing may also be a limitation of the analysis and should be commented on.

**Author Response: Thank you for the valuable feedback. We did not think about this point, and in the opinion of the reviewer, we mentioned the limitation of the manuscript. (Page 19, Line 323-325)**

The Kaplan-Meier curves both potentially strengthen and weaken their arguments. It is noteworthy that the survival time for the late-steroid group is longer than the early-steroid group, and is perhaps the strongest supporting piece of data for steroids increasing mortality. However, it is also notable that the late-steroid group appears to diverge from the non-steroid group around day 5 to 6, but the late-steroid group by definition received steroids after day 5. Thus, this divergence would not be due to steroids and suggests that matching still left the late-steroid group with sicker patients. This is likely another sign of a limitation of the paper.

**Author Response: Thank you for the valuable points. We have tried to do an in-depth analysis of the steroids treatment as much as possible using retrospective data. However, we thought there was the limitation as the reviewer mentioned because of not enough size of sample to analyze in the subgroups, so it supported the needs for well-designed prospective studies for the future. We mentioned the limitation in the manuscript. (Page 19, Line 323-325)**

This analysis is also limited by a fairly small sample size, particularly with regard to the subgroup analyses based on APACHE score.

**Author Response: Thank you for the valuable feedback. We did not think about this point, and in the opinion of the reviewer, we mentioned the limitation of the manuscript. (Page 19, Line 323-325)**

Reviewer #2: The changes suggested in the Summary and General Comments as well as other specific comments should be incorporated into the discussion where appropriate.
**Author Response: As your suggestion, we have revised our manuscript. Please refer to revised manuscript.**

Reviewer #3: The conclusions reached by the authors are broadly supported by the data as presented. The analyses performed on the propensity matched dataset are the most compelling data presented in this study, and support a conclusion that steroid therapy may lead to adverse outcomes, particularly in mild cases of SFTS. Given the importance of propensity matching to this study, it all the more important that the presented analyses are performed on the same matched dataset. The authors discuss the limitations of their study, and mention the potential for confounding by severity, as sicker patients were more likely to receive steroids and ribavirin, but this remains the largest potential weakness of the study.

**Author Response: Thank you for your comments. Since we discussed the results of analysis by propensity score matching in consideration of confounder as severity, we believed that the weakness related to this was probably offset.**

Additional comments regarding conclusions:
Line 316: I don’t think the data regarding 30d survival after steroid therapy for patients with mild disease actually appears in the paper. If it is going to be a discussion point, the data should be included.
**Author Response: We mentioned the data of 30 day survival time after steroid therapy for patients with mild disease in Figure 3B. In this result, in APACHE II score was less than 14, the 30-day survival time was 24.9 days in steroid group, which was shorter than 29.2 days in non-steroid group.**

--------------------

**Editorial and Data Presentation Modifications?**

Reviewer #1: (No Response)

Reviewer #2: Major revision required to address the above issues.

Reviewer #3: Line 54: Would capitalize ‘phlebovirus’ as the proper name of a viral genus

**Author Response: Thank you for your comments, we have revised as per your recommendation.**

Line 64: The reported CI for mean survival in patients with mild disease after steroid treatment is reported in the abstract as 24.9 (95% CI 20.21 – 28.53), but in Figure 3B it is reported as 24.9 (95% CI 21.21 – 28.53). Which is correct?
**Author Response: Thank you for pointing out. The correct 95% CI value was 21.21-28.53. We have modified the manuscript.**

Line 74: Would capitalize ‘phlebovirus’ as the proper name of a viral genus
**Author Response: Thank you for your comments, we have revised as per your recommendation.**

Line 170: The text states that of the 142 patients, 130 had confirmed data related to a tick bite. Would recommend including this information in Table 1 (would create a footnote, as was done for ‘Initial clinical manifestation’ and ‘Complications, total’.
**Author Response: Thank you for your comments, we have revised as per your recommendation. In addition, as the reviewer’s suggestion, we have added this information to supplementary table (S2 Table).**

Line 162: Would present data from non-fatal group first, followed by fatal group, to match formatting of Table 1.
**Author Response: Thank you for your comments, we have revised as per your recommendation.**

Line 171: The line ‘Of the 131 patients with a confirmed diagnosis on the basis of physical examination’ is unclear. Does that mean that only 131 patients had a physical exam performed?

**Author Response: 131 patients had checked physical exam whether or not they had eschar or trace of tick bite, and of the others’ data was unclear.**

Line 187: The line that ends with (P = .144, P = .889) should cite Table 2, as this is the first reference to that table.
**Author Response: Thank you for your comments, we have revised as per your recommendation.**

Line 190 – 192: Would reorder these therapies in order that they appear in the table.
**Author Response: Thank you for your comments, we have revised as per your recommendation.**

Line 202: Keep significant units consistent. Most AST/ALT results are reported to the nearest whole number.

**Author Response: Thank you for your comments, we have revised as per your recommendation.**

Line 202: The P value for AST is reported as 0.023 in the text, but 0.024 in Table 3.

**Author response: Thank you for pointing out. The correct value was 0.024. We have modified the manuscript.**

Figure 2A: Should include a column for ‘n’ in this figure (similar to other figures)

**Author Response: Thank you for your comments, we have revised as per your recommendation. We have also added the information of missing data to a footnote of the figure2A.**

Line 307-308: Unclear what this sentence regarding ribavirin contributes to the discussion or to the analysis overall.
**Author Response: Thank you for pointing out. We decided that the sentence was not relevant to this paper and deleted it.**

--------------------

**Summary and General Comments**


Use this section to provide overall comments, discuss strengths/weaknesses of the study, novelty, significance, general execution and scholarship. You may also include additional comments for the author, including concerns about dual publication, research ethics, or publication ethics. If requesting major revision, please articulate the new experiments that are needed.

Reviewer #1: The authors undertook a retrospective analysis using propensity score matching to attempt to achieve a measure of equipoise in comparing steroids to no steroids in the treatment of SFTS given a lack of data comparing this. As with any retrospective analysis, there are limitations that restrict how strong of a conclusion can be drawn. In particular, the range of steroid dosing appears to be quite wide, and there is a suggestion from the KM curves that those who received steroids were still sicker than those who did not. Presumably, from the wording of the conclusion section, the increased complications in the steroid group occurred after steroids were given, but this is not apparent from the results section and should be clarified. Regardless, there is a suggestion of harm with steroids, and clinicians should carefully consider whether to administer them in patients with SFTS until better data is available.
**Author Response: We appreciate of your valuable comments. We agree with the reviewer’s opinion. In the result section, regarding the increase in complications after steroid administration, we have tried to explain more clearly by modifying the result of analysis using the dataset after propensity score matching. Please refer to the revised manuscript.**

Reviewer #2: The authors evaluated the effects of steroid therapy on Korean patients hospitalized at multiple centers with documented Severe Fever with Thrombocytopenia Syndrome (SFTS), a potentially fatal tick-borne viral infection. To address potential indication bias in this retrospective study, they performed propensity score matching and showed that mortality was significantly higher in steroid-treated patients who were either less severely ill (low APACHE II) or who were treated early (<5 days after symptom onset). Therefore, they recommend caution when using steroids for SFTS.
**Author Response:** **We appreciate of your valuable comments and explaining the strengths of our research.**

This is a well conducted study with rigorous statistical analyses and appropriate adjustments for potential biases. There are no other published studies that have addressed the utility and complications of steroid use for SFTS. Therefore, within its limitations as outlined by the authors, this study may be useful for clinicians where the disease is prevalent (Korea, China and Japan).
**Author Response:** **We appreciate of your valuable comments and explaining the strengths of our research. We completely agree with the reviewer’s opinion as the importance of useful this study’s results.**

However, there are major weaknesses that should be addressed:
1) It appears that the rate of SFTS increased from 2013 to 2017 (Figure 1) but it is unclear if this trend may have been associated with increased testing (other possible reasons could have been an epidemic or increased virulence of the virus). The authors should present the total number of patients tested and the proportion of positive tests each year during that time period.
2) The potential effect of unidentified co-infections: (pg 6, line 148) “no patient had a confirmed co-infection”. What other infections were tested, were they routinely tested and what proportion of patients were tested? This should be added.
3) It is not clear what the distribution of “time to start of steroids” was in patients who survived vs those who died –this should be added to Table 1.
4) How do the authors explain that frequencies of certain clinical variables were lower in the steroid group vs the non-steroid group (Table 2) such as: fever, chills, myalgia and fatigue although the APACHE II score was higher? This should be discussed.
5) The Glasgow Coma Scale was lower after steroid treatment (Table 3) which should be commented on in the discussion in the context of the paper they cite (Nakamura et al) that recommended steroids for SFTS-associated encephalopathy.
6) On pg 14 (Table 4), the authors describe risk factors for 30-day mortality but most of these are later events and complications (eg ICU admission, mechanical ventilator, hemodialysis) and are not contextualized within the relevant literature describing prediction models that they have not cited such as:
a. He et al. Severe fever with thrombocytopenia syndrome: a systematic review and meta-analysis of epidemiology, clinical signs, routine laboratory diagnosis, risk factors, and outcomes. BMC Infect Dis. 2020; 20: 575
b. Wang et al. A nomogram to predict mortality in patients with severe fever with thrombocytopenia syndrome at the early stage - A multicenter study in China. PLoS Negl Trop Dis. 2019 Nov; 13(11): e0007829
c. Liu et al. Analysis of the laboratory indexes and risk factors in 189 cases of severe fever with thrombocytopenia syndrome. Medicine (Baltimore). 2020 Jan;99(2):e18727.
d. Li et al. Epidemiological and clinical features of laboratory-diagnosed severe fever with thrombocytopenia syndrome in China, 2011–17: a prospective observational study. Lancet Infect Dis. 2018 Oct;18(10):1127-1137.
7) The authors compared the rates of complications in the steroid vs non-steroid groups (Table 7) but they did not adjust for severity and potential indication bias as they did in Table 6. Therefore, some of these complications may have been related to severe disease and not steroids. Propensity score matching should be added and the discussion should be modified.

**Author Response: Thank you for your valuable comments. We would like to explain the weakness in our study mentioned by the reviewer as follows.**

**1) According to the point out, we have indicated the data of the total number of patients tested in Figure 1.**

**2) There were different pathogen types tested by routines between hospitals. However, tests were conducted to identify the pathogens of tick-borne diseases or vector-borne diseases such as scrub typhus (Orientia tsutsugamushi), leptospirosis(Leptospira), and hemorrhagic fever with renal syndrome (Hantaan virus) in common with each hospitals. Also, of the 142 participants in this study, 51 patients whose samples were available were tested for scrub typhus and HFRS by PCR, and all of them were the results of negative.**

**3) According to the point out, We have added “Days from admission to first treatment (of steroids)” variable to Table 1 for the distribution of time to steroid start.**

**4) We have found the reason for the different results of APACHE II score and the patient’s symptoms as that the symptoms were subjective data from the patients, and as the patient’s condition was severe especially in altered mental status, the patients did not express the subjective symptoms.**

**Therefore, we have tried to consider and analyzed not only the patient’s subjective symptoms, but also the variables of objective data such as APACHE II score and laboratory data. As the reviewer pointed out, we described it in the discussion section.**

**5)According to the comment the reviewer pointed out, we have added the relevant part in the discussion section (Page 17, Line 281).**

**6) We found out difference between non-fatal and fatal groups through univariate analysis of all variables corresponding to epidemiological, initial symptoms, and initial laboratory data in Table 1 and 2 in addition to the variables in Table 4 as risk factors for 30-day mortality. Among them, the risk factors of 30-day mortality were confirmed by performing multivariate analysis using variables included in Table 4 as variables as p value < .1 in univariate analysis. AUC value of the multivariate model in Table 4 was 0.909.**

**7) According to the reviewer’s comments, we revised Table 7 and the related sentences as the results of analysis using the dataset after propensity score matching (Page 16, Line261-265).**

Reviewer #3: Overall, this study presents novel findings that may inform treatment for a significant emerging pathogen, and is thus of importance from both a clinical and public health standpoint. The primary strength of this study is novelty, and the incorporation of a number of SFTS patients from several centers over multiple years potentially makes it more generalizable. However, the number of patients included in this study is still relatively small, and with any retrospective cohort study, it is difficult to identify and control for all potential confounding variables. The possibility that patients receiving steroids are more sick, or at a different point in their illness, leading to both poorer outcomes and making it more likely that they will receive steroids, is the biggest potential source of error. Still, the novelty and potential clinical impact of this study are of interest, and warrant publication with minor revisions.

**Author Response: We appreciate of the valuable comments. In the case of the possibility that patients receiving steroids were more sick, we believe that it may have been controlled through propensity score matching using the variables such as APACHE II score. In the case of the possibility that patients at a different point in their illness, as shown in Table 2 results, there was no statistical difference in ‘symptom onset to admission’ between steroids and non-steroids groups, so we decided that the possibility was small. For the minor revisions checked by the reviewer, we have revised manuscript.**
