# Supplementary material for: Effects of steroid therapy in patients with severe fever with Thrombocytopenia syndrome: A multicenter clinical cohort study
Source: PLoS Negl Trop Dis. 2021 Feb 19;15(2):e0009128. doi: 10.1371/journal.pntd.0009128 (PMC7928499; doi:10.1371/journal.pntd.0009128)
Supplement: Attachment — Submitted filename: Response letter PNTD-D-20-01574R1.docx [file pntd.0009128.s004.docx]

29^th^ December 2020

A. Desiree LaBeaud - Deputy Editor
Anita K. McElroy, MD, PhD - Associate Editor
*PLOS Neglected Tropical Diseases*

Re: PLOS Neglected Tropical Diseases – PNTD-D-20-01574R1

Dear Editors,

I, along with my co-authors, would like to re-submit the attached manuscript entitled “Effects of Steroid Therapy in Patients with Severe Fever with Thrombocytopenia Syndrome: A Multicenter Clinical Cohort Study”. The manuscript ID is **PNTD-D-20-01574R1**.

We would like to thank all the reviewers and editors for taking the time to review and are grateful for their constructive comments and valuable improvements to our manuscript. In response to the Editors comment, here with this file I had attached the point-by-point response comments in bold. All page numbers refer to the revised manuscript file without track changes. We have addressed all the raised concerns, and believe that by making these changes the quality of the manuscript has been substantially improved.

With best wishes

**Dong-Min Kim**

Department of Internal Medicine, College of Medicine, Chosun University

588 Seosuk-dong, Dong-gu, Gwangju 61453, Republic of Korea

Tel: 82-62-220-3108; Fax: 82-62-234-9653
**Responses to Reviewer's comment.**

**Methods**

Reviewer #1: I had no prior concerns about the methods and this remains the case
**Author response: Thank you for the reviewer’s comment.**

Reviewer #2: The authors explained the inclusion criteria in their response to reviewers but need to include it in the Methods section
**Author response: Thank you for your comment. We have added the inclusion criteria on manuscript as per your recommendation (page 5, Line 104-105).**

--------------------
**Results**

Reviewer #1: I appreciate that the authors have provided further information regarding steroid dosing in Table 2 with median and IQR, as this certainly gives a better sense than what is described in the body of the paper.  I think adding this information in Table 1 for the non-fatal and fatal groups would also be useful though in order to have some sense as to whether those that died received a different sort of dosing regimen than those that survived.
**Author response: Thank you for the positive feedback. We have added the data of steroid dose in Table 1 as per your recommendation.**

For Table 7, in their response regarding timing of complications relative to timing steroid dosing, they stated that due to the retrospective nature of the study, they had difficulty in determining timing of complications.  This is understandable, but then this uncertainty should be made explicitly clear in the paper.
**Author response: Thank you for the valuable feedback. As your suggestion, we mentioned on the manuscript (Page 19, Line 320-322).**

Reviewer #2: Overall much improved
The methods of testing (PCR and serology) were explained in their response to reviewers but were not included in the text.

**Author response: Thank you for your recommends. As your suggestion, we have revised our manuscript (page 6, Line 143-145).**

The authors explained in their responses that the steroid dosages and types of steroids used differed significantly among centers but they need to add a comment in the text or as a footnote
**Author response: Thank you for your valuable comments. We have added the information as a footnote in Table 1 as per your suggestion.**

--------------------

**Conclusions**

Reviewer #1: Following the point about timing of complications in results, the authors on pg 19, lines 328-329 state "In our study we observed an increase in various complications following steroid therapy"; there is a similar sentence on line 338.  However, their response mentioned above seems to suggest that they were unable to actually assess the timing of most complications relative to steroid therapy.  Thus, if they wish to point out the increase in complications, it should read something to the effect of "an increase in various complications in those who received steroid therapy".
**Author response: Thank you for your valuable feedback.** **As your recommends, we have revised the point on the manuscript (Page 19, Line 320).**

Otherwise, I believe their conclusions are adequately supported by the results.  The most significant limitations of the analysis are beyond the control of the authors, namely sample size and heterogeneity of practice, but as they state, a prospective analysis would be needed to address this.  Thus, demonstrating the uncertainty of the role of steroids and need for prospective study is perhaps the most important conclusion.
**Author response: We appreciate for pointing out important things. We agree with the reviewer comments. We have added the points in conclusion on the manuscript (Page 19, Line 333-334).**

Reviewer #2: Limitations are explained better
**Author response: We appreciate for your comment.**

--------------------

**Editorial and Data Presentation Modifications?**
Use  this section for editorial suggestions as well as relatively minor modifications of existing data that would enhance clarity. If the only modifications needed are minor and/or editorial, you may wish to recommend “Minor Revision” or “Accept”.

Reviewer #1: Minor revision and would endorse accepting after the above changes are made.

Reviewer #2: (No Response)

--------------------

**Summary and General Comments**


Reviewer #1: The authors have incorporated most of the feedback previously provided and I believe the paper is stronger as a result.  With a few small modifications and clarifications, I believe it would be suitable for publication.  Although its small sample size and retrospective nature is certainly not enough to base practice on, it does provide some suggestion of worse outcome with steroids, in contradiction to other published results in support of steroids, and thus as the authors state, supports the need for prospective study of the subject.
 **Author response: We appreciate your valuable feedback, which helped us to improve our manuscript. We have impressed with the valuable comments, and had updated the conclusion according to the reviewer’s points.**

Reviewer #2: I am satisfied with the authors responses overall
**Author response: We appreciate your positive feedback. We have impressed with the valuable comments.**
